# Sirtuin 3 deficiency does not alter host defenses against bacterial and fungal infections

**DOI:** 10.1038/s41598-017-04263-x

**Published:** 2017-06-20

**Authors:** Eleonora Ciarlo, Tytti Heinonen, Jérôme Lugrin, Hans Acha-Orbea, Didier Le Roy, Johan Auwerx, Thierry Roger

**Affiliations:** 1 Infectious Diseases Service, Department of Medicine, Lausanne University Hospital, CH-1066 Epalinges, Switzerland; 2 Department of Biochemistry, University of Lausanne, CH-1066 Epalinges, Switzerland; 3 Laboratory for Integrative and Systems Physiology, Ecole Polytechnique Fédérale de Lausanne, CH-1015 Lausanne, Switzerland

## Abstract

Sirtuin 3 (SIRT3) is the main mitochondrial deacetylase. SIRT3 regulates cell metabolism and redox homeostasis, and protects from aging and age-associated pathologies. SIRT3 may drive both oncogenic and tumor-suppressive effects. SIRT3 deficiency has been reported to promote chronic inflammation-related disorders, but whether SIRT3 impacts on innate immune responses and host defenses against infections remains essentially unknown. This aspect is of primary importance considering the great interest in developing SIRT3-targeted therapies. Using SIRT3 knockout mice, we show that SIRT3 deficiency does not affect immune cell development and microbial ligand-induced proliferation and cytokine production by splenocytes, macrophages and dendritic cells. Going well along with these observations, SIRT3 deficiency has no major impact on cytokine production, bacterial burden and survival of mice subjected to endotoxemia, *Escherichia coli* peritonitis, *Klebsiella pneumoniae* pneumonia, listeriosis and candidiasis of diverse severity. These data suggest that SIRT3 is not critical to fight infections and support the safety of SIRT3-directed therapies based on SIRT3 activators or inhibitors for treating metabolic, oncologic and neurodegenerative diseases without putting patients at risk of infection.

## Introduction

The innate immune system provides the first line of defense against microbial infections. Innate immune cells such as macrophages and dendritic cells (DCs) detect invading microorganisms through pattern recognition receptors (PRRs). The best-characterized family of PRRs is constituted by Toll-like receptors (TLRs), which mediate the sensing of a broad range of microbial structures^1^. The interaction between PRRs and microbial ligands activates intracellular signaling pathways that coordinate the expression of immune-regulatory genes among which cytokines/chemokines, and the development of humoral and cellular responses required to neutralize or eliminate pathogens and restore homeostasis.

Sirtuins (SIRT1–7) belong to the NAD^+^-dependent class III subfamily of histone deacetylases (HDACs)^2^. Besides histones, sirtuins target thousands of non-histone proteins, among which chromatin modifiers, transcription regulators, signal transduction molecules, metabolic enzymes and structural cell components^3^. SIRT1–7 localize in the cytosol, nucleus and/or mitochondria, which dictates their accessibility to substrates and effector functions.

SIRT3 is the main mitochondrial deacetylase^4, 5^. SIRT3 concentrates primarily to the matrix of the mitochondria but may also localize into the nucleus^6, 7^. SIRT3 deacetylase activity is intrinsically linked to cell metabolism^8^. SIRT3 promotes fatty acid β-oxidation, tricarboxylic acid cycle, ketogenesis, urea cycle and brown adipose tissue thermogenesis^9–15^. SIRT3 also regulates the activity of the electron transport chain and dampens oxidative stress by targeting superoxide dismutase 2 and the glutathione system^16^. As a regulator of metabolism and oxidative stress homeostasis, SIRT3 protects from aging and age-associated dysfunctions, and genetic studies identified *SIRT3* polymorphisms associated with increased longevity^17–20^.

SIRT3 protects from stress-induced cardiovascular diseases and impacts on the development of neurodegenerative and oncologic diseases^21–28^. SIRT3 deficiency increases allograft graft injury, diabetic cardiac dysfunction, insulin resistance, acute kidney injury and lung fibrosis^29–38^, suggesting that SIRT3 may counteract the development of chronic metabolic and inflammation-related disorders. SIRT3 has been reported to drive oncogenic and tumor-suppressive effects^39^. All these observations stimulated the development of both activators and inhibitors of SIRT3 for clinical purposes^40^. Within this context, it is important to ascertain that SIRT3 targeting would not negatively impact on host resistance to infection, an aspect of SIRT3 biology that is so far poorly characterized^41, 42^.

In the present study, we used SIRT3 knockout mice to investigate whether SIRT3 deficiency altered the response of immune cells to microbial ligands *in vitro*. We then analyzed the impact of SIRT3 deficiency in a panel of severe and non-severe models of endotoxemia, peritonitis, pneumonia, listeriosis and candidiasis. Overall, our results suggest that SIRT3 deficiency has no major impact on host defenses against infections, supporting the safety of SIRT3-oriented therapies currently under development.

## Materials and Methods

### Ethics statement

Animal experimentations were approved by the Service de la Consommation et des Affaires Vétérinaires (SCAV) du Canton de Vaud (Epalinges, Switzerland) under authorizations n° 876.8, and 877.8, and performed according to Swiss guidelines and ARRIVE guidelines (http://www.nc3rs.org.uk/arrive-guidelines).

### Mice, cells and reagents

*Sirt3* floxed (*Sirt3* ^*L2/L2*^) mice were generated as described^43^ and crossed with mice expressing the CMV-Cre deleter in the male germline to create full knockouts. SIRT3^−/−^ mice were backcrossed nine times on a C57BL6/J background. Mice were housed under specific pathogen-free conditions and were exempt of mouse hepatitis virus and murine norovirus infections. Splenocytes were cultured in RPMI 1640 medium containing 2 mM glutamine, 50 μM 2-ME, 100 IU/ml penicillin, 100 μg/ml streptomycin (Invitrogen, San Diego, CA) and 10% heat-inactivated fetal calf serum (FCS, Sigma-Aldrich St. Louis, MO)^44^. Bone marrow (BM) cells were cultured seven days in IMDM (Invitrogen) containing 2-ME, penicillin, streptomycin, 10% FCS and 50 U/ml macrophage colony-stimulating factor or 250 U/ml granulocyte macrophage colony-stimulating factor (Immunotools, Friesoythe, Germany) to generate BM-derived macrophages (BMDMs) and dendritic cells (BMDCs)^45^. For experiments, 10^5^, 5 × 10^5^ or 2 × 10^6^ cells were seeded in 96, 24 or 6-well plates in complete medium without growth factor and antibiotics. The stimuli used were: *Salmonella minnesota* ultra pure LPS (List Biologicals Laboratories, Campbell, CA), Pam_3_CSK_4_ (EMC microcollections, Tübingen, Germany), CpG ODN 1826 (CpG, InvivoGen, San Diego, CA), concanavalin A (Sigma-Aldrich), anti-CD3ε and CD28 antibodies (clones 145-2C11 and 37.51, eBioscience, San Diego, CA) and toxic shock syndrome toxin-1 (TSST-1, Toxin Technology, Sarasota, FL). Clinical strains of *Escherichia coli* O18 (*E*. *coli*), *Klebsiella pneumoniae* caroli (*K*. *pneumoniae*), Group B Streptococcus (GBS)^46–49^ and *Listeria monocytogenes* 10403 s (*L*. *monocytogenes*, a gift from D. Zehn, Lausanne University Hospital, Switzerland) were grown in brain heart infusion broth (BD Biosciences, Erembodegem, Belgium). *Candida albicans* 5102 (*C*. *albicans*)^44^ was cultured in yeast extract-peptone-dextrose (BD Biosciences). Bacteria were heat-inactivated for *in vitro* experiments^50^.

### RNA analyses

RNA was isolated and reverse transcribed using the RNeasy and QuantiTect reverse transcription kits (Qiagen, Hilden, Germany). Real-time PCR was conducted using the Fast SYBR® Green Master Mix and a QuantStudio™ 12 K Flex system (Life Technologies, Carlsbad, CA)^44, 45^. Primers have been described^45, 51^. Sirt3 expression was normalized to actin expression. Sirt3 mRNA expression levels in organs were extracted from the BioGPS resource (http://biogps.org).

### Western blot analyses

Proteins were extracted from liver or BMDMs using RIPA lysis buffer (150 mM NaCl, 50 mM Tris-HCl pH 7.4, 1 mM EDTA, 1% Triton-X-100, 0.1% NP-40, 1 mM PMSF) or an in house cell lysis buffer (150 mM NaCl, 10 mM Tris-HCl pH 7.5, 1 mM EDTA, 0.5% NP-40, 1 mM PMSF, 1 mM Na-orthovanadate, 10 mM NaF) containing protease and phosphatase inhibitors (Merck)^52, 53^ and electrophoresed through SDS-PAGE^54^. Membranes were incubated with antibodies directed against SIRT3 and total and phosphorylated ERK1/2, p38 and JNK (Cell Signaling Technology), then with a secondary horseradish peroxidase-conjugated antibody (Sigma-Aldrich). Blots were revealed with the enhanced chemiluminescence Western blotting system (GE Healthcare, Little Chalfont, Royaume-Uni). Images were recorded using a Fusion Fx system (Viber Lourmat, Collégien, France).

### Flow cytometry

Single cell suspensions from thymus and spleen were incubated with 2.4G2 monoclonal antibody (mAb) and stained using mAbs listed in Supplementary Table S1 as described previously^55^. Data were acquired using a LSR II flow cytometer (BD Biosciences) and analyzed using FlowJo Version 8.5.3 software (FlowJo LLC, Ashland OR).

### Proliferation assay

Splenocytes were cultured in 96-well plates for 48 hours with different stimuli and proliferation quantified by measuring ^3^H-thymidine incorporation over 18 hours^56^.

### Cytokine measurements

Cytokines were quantified using DuoSet ELISA kits (R&D Systems, Abingdon, UK)^57^.

### *In vivo* models

Eight to twelve-week-old SIRT3^+/+^ and SIRT3^−/−^ C57BL/6 J mice matched for age were used. Endotoxemia was performed by challenging mice intraperitoneally (i.p.) with 400 μg LPS (20 mg/kg). Peritonitis, pneumonia, listeriosis and candidiasis were induced by injecting i.p. 1–3 × 10^4^ CFU *E*. *coli*, intranasally (i.n.) 30 CFU *K*. *pneumoniae*, intravenously (i.v.) 10^5^ CFU *L*. *monocytogenes* and i.v. 10^5^, 3 × 10^5^ or 10^6^ CFU *C*. *albicans*, respectively. Blood, spleen and liver were collected 1–48 hours post-challenge to quantify cytokines and bacteria. Survival and severity scores graded from 1 to 5 were registered at least once daily^58^. Animals were euthanized when they met a severity score of 4. Animal follow-up was performed by two operators.

### Statistical analyses

Comparisons between groups were performed using the ANOVA F-test followed by two-tailed unpaired Student’s t-test. Survival curves were built using the Kaplan-Meier method and differences were analyzed by the log-rank sum test. All analyses were performed using PRISM (GraphPad, San Diego, CA). *P* values were two-sided and significance level was set at 0.05.

## Results

### SIRT3 deficiency has no major impact on the composition of thymic and splenic immune cell subsets and on the response of splenocytes to immune stimuli

**Figure 1:**
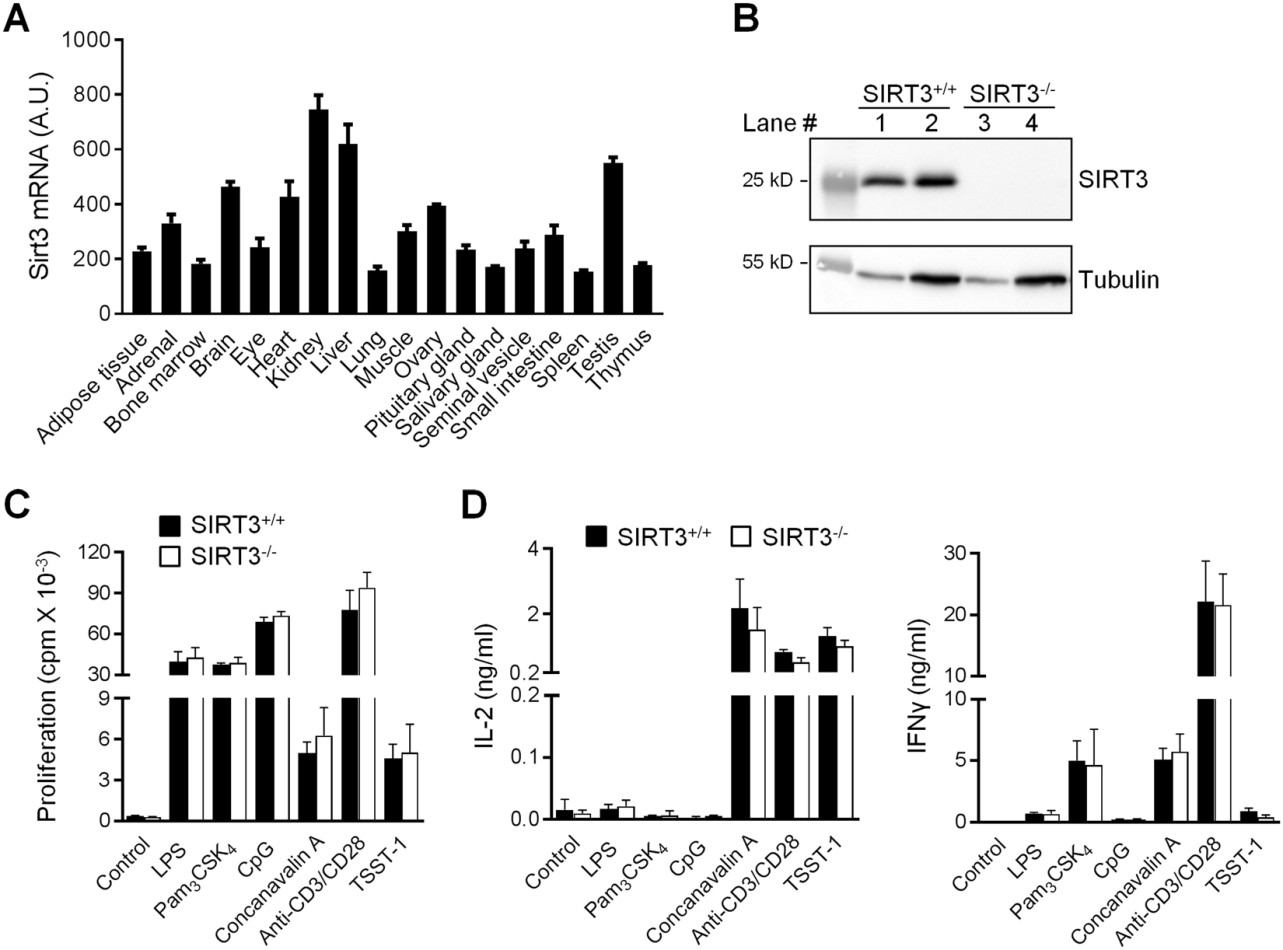
SIRT3 deficiency does not influence proliferation and cytokine response of splenocytes. (**A**) Sirt3 mRNA expression levels in a panel of organs (from the BioGPS resource). A.U.: arbitrary units. (**B**) SIRT3 protein expression in SIRT3^+/+^ and SIRT3^−/−^ liver (protein extracts from lanes 1 and 3 and lanes 2 and 4 were obtained using RIPA lysis buffer and in house cell lysis buffer, respectively) assessed by Western blotting. Full-length blots are presented in Supplementary Figure S1. The first lane of each blot corresponds to a molecular weight marker. (**C**,**D**) SIRT3^+/+^ and SIRT3^−/−^ splenocytes were incubated for 48 hours with LPS (5 µg/ml), Pam_3_CSK_4_ (10 µg/ml), CpG (2 µg/ml), concanavalin A (5 µg/ml), anti-CD3/CD28 antibodies (1 µg/ml) and TSST-1 (2 µg/ml). (**C**) Proliferation was measured by ^3^H-thymidine incorporation. Data are means ± SD of triplicate samples from one experiment performed with 3 mice and are representative of 2 experiments. (**D**) IL-2 and IFNγ concentrations in cell culture supernatants were quantified by ELISA. Data are means ± SD from two experiments each performed with 3 mice.

SIRT3 is expressed ubiquitously, including in immune organs (Fig. 1A). SIRT3^−/−^ mice used in this study were backcrossed nine times on a C57BL6/J background. SIRT3^−/−^ mice developed without macroscopic abnormalities and expressed no detectable levels of SIRT3 protein (Fig. 1B). The absolute number of cells and the proportions of the major immune cell subsets in the thymus and the spleen were similar in SIRT3^+/+^ and SIRT3^−/−^ mice, including CD4/CD8 double negative (DN1-4), double positive (DP) and single positive (SP) thymocytes and splenic CD3^+^ T cells (DN and SP, naïve and memory), B220^+^ B cells (immature and mature) and CD11c^+^ DCs (Table 1 and Table 2). SIRT3^+/+^ and SIRT3^−/−^ splenocytes were cultured for 48 hours with LPS (TLR4 ligand), Pam_3_CSK_4_ (TLR1/TLR2 ligand), CpG (TLR9 ligand), concanavalin A, anti-CD3/CD28 and TSST-1 before measuring cell proliferation and IL-2 and IFNγ production. SIRT3^+/+^ and SIRT3^−/−^ splenocytes reacted similarly to all stimuli (Fig. 1C and D). Hence, SIRT3 deficiency had no apparent impact on immune cell development and splenocyte response to stimulation.

**Table 1:** Thymic cell subsets in SIRT3^+/+^ and SIRT3^−/−^ mice.

	SIRT3^+/+^	SIRT3^−/−^
CD4^+^ CD8^+^	82.3 ± 3.1	84.3 ± 1.0
CD4^−^ CD8^−^	2.0 ± 0.6	1.6 ± 0.1
CD25^−^ CD44^+^	1.4 ± 0.6	1.2 ± 0.2
CD25^+^ CD44^+^	0.1 ± 0.01	0.1 ± 0.03
CD25^+^ CD44^−^	1.8 ± 0.6	1.8 ± 0.3
CD25^−^ CD44^−^	96.6 ± 1.2	96.8 ± 0.42
CD4^+^ CD8^−^	12.0 ± 2.3	10.6 ± 0.7
CD4^−^ CD8^+^	3.6 ± 0.3	3.5 ± 0.2

Data are means ± SD of 4 animals per group and expressed as the percentage of total cells (CD4^+^ CD8^+^, CD4^−^ CD8^−^, CD4^+^ CD8^−^ and CD4^−^ CD8^+^) or percentage of CD4^−^ CD8^−^ parental cells (CD25^−^ CD44^+^, CD25^+^ CD44^+^, CD25^+^ CD44^−^ and CD25^−^ CD44^−^). Total cell numbers were 49.2 ± 15.4 and 47.4 ± 6.9 millions per thymus in SIRT3^+/+^ and SIRT3^−/−^ mice, respectively. No statistically significant differences in subsets’ percentages and absolute numbers were detected.

**Table 2:** Splenic cell subsets in SIRT3^+/+^ and SIRT3^−/−^ mice.

	SIRT3^+/+^	SIRT3^−/−^
CD3^+^ T cells (%)	27.3 ± 4.6	31.1 ± 1.0
CD4^+^	62.3 ± 2.7	61.2 ± 4.2
CD4^+^ CD44^low^ CD62L^high^ (naive)	46.0 ± 4.2	48.9 ± 6.8
CD4^+^ CD44^high^ CD62L^low^ (memory)	16.2 ± 2.6	12.0 ± 2.8
CD8^+^	31.5 ± 2.0	32.5 ± 3.3
CD8^+^ CD44^low^ CD62L^high^ (naive)	23.3 ± 1.0	28.6 ± 1.7
CD8^+^ CD44^high^ CD62L^low^ (memory)	8.2 ± 0.5	6.6 ± 0.3
CD4^−^ CD8^−^	1.3 ± 0.1	1.5 ± 0.3
B220^+^ B cells (%)	50.5 ± 7.6	53.3 ± 3.1
B220^+^ IgD^+^ CD23^+^ (mature)	45.7 ± 2.6	48.8 ± 2.7
B220^+^, non-IgD^+^/CD23^+^ (immature)	6.5 ± 0.5	6.4 ± 0.5
CD11c^+^ DCs (%)	6.2 ± 0.5	6.5 ± 0.6
B220^−^	62.8 ± 2.5	59.4 ± 2.6
B220^+^	37.2 ± 2.5	40.6 ± 2.6

Data are means ± SD of 4 animals per group and expressed as the percentage of CD3^+^, B220^+^, and CD11c^+^ splenic cells or the percentage of the CD3^+^, B220^+^ and CD11c^+^ parental populations expressing CD4, CD8, CD44, CD62L, IgD and CD23. Total cell numbers were 75.1 ± 7.5 and 75.4 ± 21.1 millions per spleen in SIRT3^+/+^ and SIRT3^−/−^ mice, respectively. No statistically significant differences in subset’s percentages and absolute numbers were detected.

### SIRT3 deficiency does not alter macrophage and dendritic cell response to microbial products

**Figure 2:**
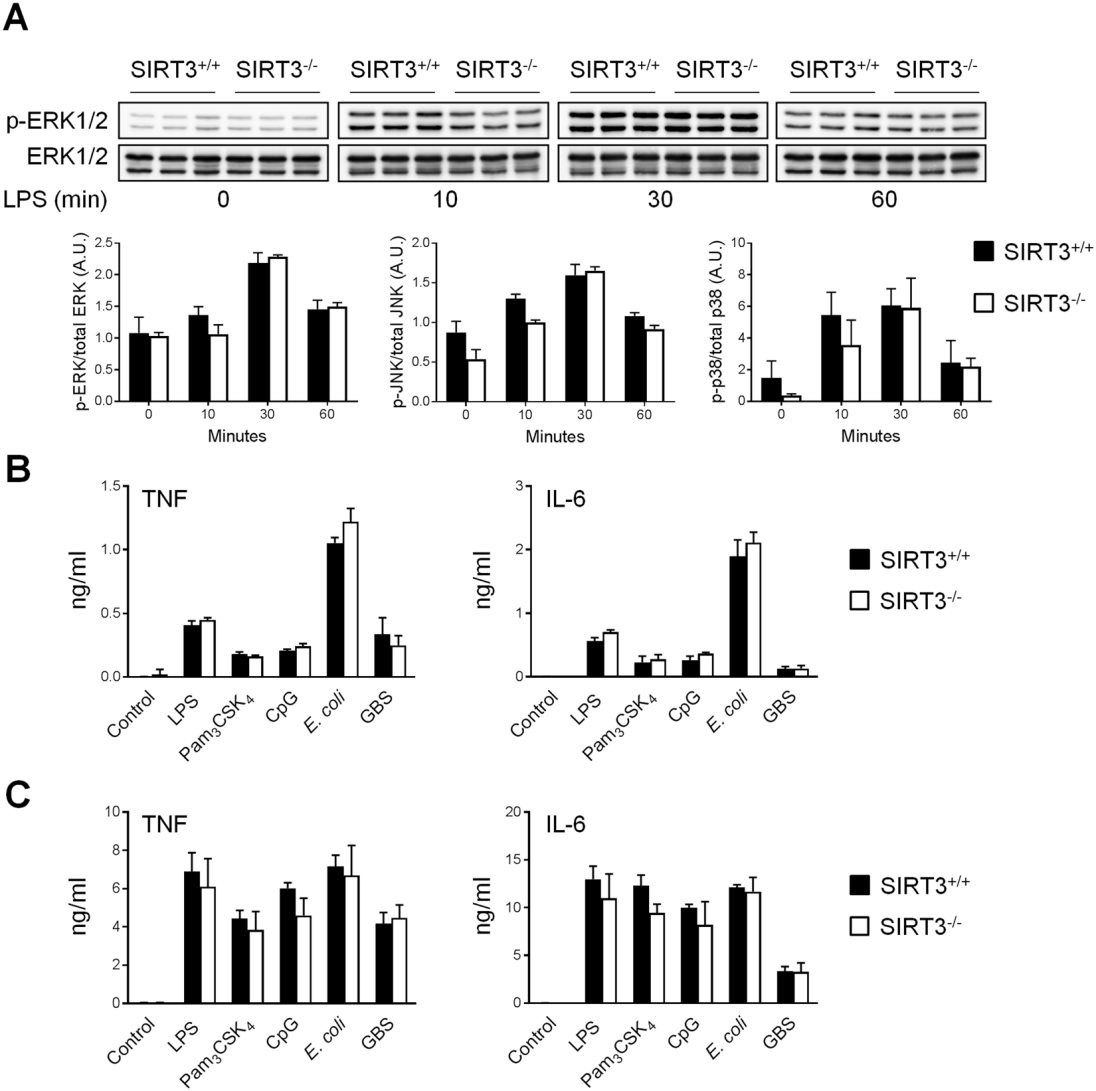
SIRT3 does not affect TNF and IL-6 production by BMDMs and BMDCs exposed to microbial stimuli. SIRT3^+/+^ and SIRT3^−/−^ BMDMs (**A**,**B**) and BMDCs (**C**) were exposed to LPS (10 ng/ml), Pam_3_CSK_4_ (10 ng/ml), CpG (2 µg/ml), *E*. *coli* (10^6^ CFU/ml) and GBS (2.5 × 10^6^ CFU/ml). (**A**) Expression levels of phosphorylated (p) and total ERK1/2, JNK and p38 were analyzed by Western blotting and quantified by imaging. Data are means ± SD obtained with 3 mice. Full-length blots are presented in Supplementary Figure S2. (**B**,**C**) TNF and IL-6 concentrations in cell culture supernatants collected 8 hours after stimulation. Data are means ± SD of triplicate samples from one experiment performed with 3 mice and are representative of 2 experiments.

TLR triggering activates the MAPK pathway involved in the control of cytokine production^1^. To test whether SIRT3 had an impact on the response of macrophages to TLR ligands, the phosphorylation of ERK1/2, p38 and JNK MAPKs in BMDMs exposed for 0, 10, 30 and 60 minutes to LPS was analyzed by Western blotting (Fig. 2A). ERK1/2, p38 and JNK phosphorylation was reduced in SIRT3^−/−^ BMDMs 10 minutes post-stimulation (Fig. 2A). No differences were observed 30 and 60 minutes post-stimulation. Nonetheless, SIRT3^+/+^ and SIRT3^−/−^ BMDMs secreted comparable levels of TNF and IL-6 (t = 8 hours) in response to stimulation with LPS, Pam_3_CSK_4_, CpG, *E*. *coli* and GBS (Fig. 2B). SIRT3^+/+^ and SIRT3^−/−^ BMDCs also produced identical levels of TNF and IL-6 when exposed to the same panel of stimuli (Fig. 2C). In agreement with these observations, SIRT3^+/+^ and SIRT3^−/−^ BMDMs expressed similar mRNA levels of a selection of PRRs (Tlr1, Tlr2, Tlr6, Tlr9, Cd14, Md2, Msr1) and cytokines/chemokines (Il1a, Il1b, Il10, Il12b, Il15, Il27, Ccl2/Mcp1, Ccl3/Mip1a, Ccl4/Mip1b, Cxcl1/Groa, Cxcl10/Ip10, Cxcl11/Itac) at baseline and 2 hours following stimulation with LPS (Supplementary Figure S3). Altogether, these results suggested that SIRT3 deficiency marginally influenced MAPK signaling and cytokine production by macrophages and DCs exposed to TLR ligands and whole bacteria.

### SIRT3 deficiency does not affect the course of endotoxemia and bacterial and fungal infections

**Figure 3:**
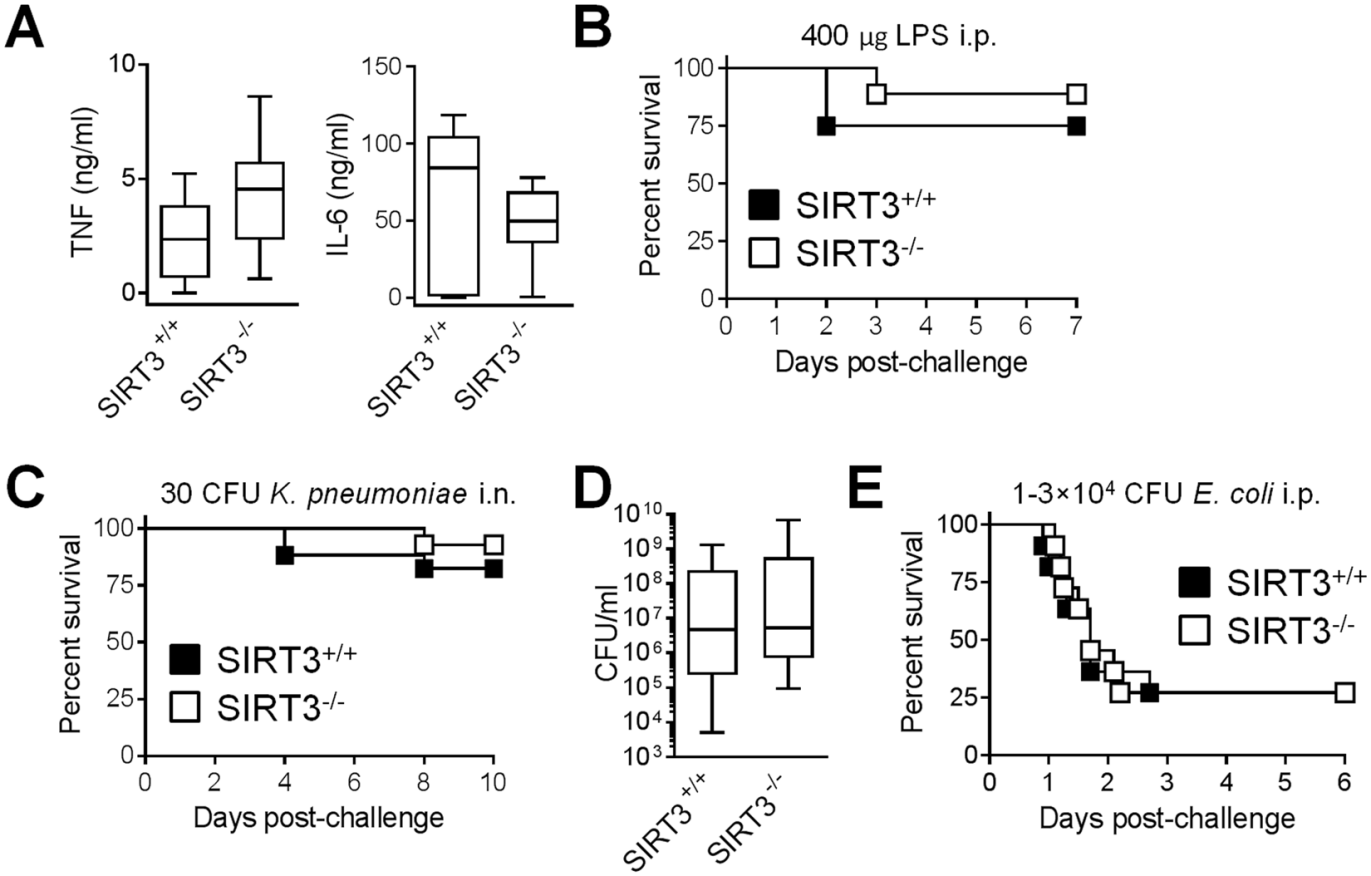
SIRT3 deficiency does not affect the course of endotoxemia and bacterial infection. SIRT3^+/+^ and SIRT3^−/−^ mice were injected i.p. with 400 μg LPS ((**A**,**B**) n = 8–9 per group), i.n. with 30 CFU *K*. *pneumoniae* ((**C**) n = 17 SIRT3^+/+^ and 14 SIRT3^−/−^ mice) and i.p. with 1–3 × 10^4^ CFU *E*. *coli* ((**D**,**E**) n = 11 per group). (**A**) TNF and IL-6 concentrations in blood collected 1 hour (TNF) and 6 hours (IL-6) after LPS challenge. P = 0.1 and 0.2. (**B**,**C** and **E**) Survival of mice. P = 0.4, 0.4 and 1.0 (**D**) CFU counts in blood collected 18 hours after *E*. *coli* challenge. P = 0.4.

To address the relevance of our *in vitro* findings, we developed a model of endotoxemia by challenging mice i.p. with 20 mg/kg LPS (Fig. 3A and B). TNF and IL-12p40 concentrations in blood collected from SIRT3^+/+^ and SIRT3^−/−^ mice 1 hour (TNF) and 6 hours (IL-6) post-challenge were comparable (Fig. 3A). In line with these results, the overall survival of SIRT3^+/+^ and SIRT3^−/−^ mice was similar (75% *vs* 89%, P = 0.4, Fig. 3B).

**Figure 4:**
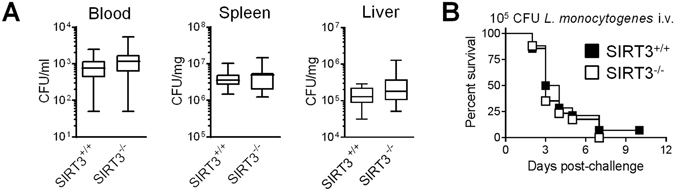
SIRT3 deficiency does not impact on organ colonization and survival of mice infected with *L*. *monocytogenes*. SIRT3^+/+^ and SIRT3^−/−^ mice (**A**) n = 14–15 per group; (**B**) n = 14–16 per group) were injected i.v. with 10^5^ CFU *L*. *monocytogenes*. ((**A**) Blood, spleen and liver were collected 48 hours after challenge to quantify bacterial loads. P = 0.02, 0.8 and 0.1. (**B**) Survival of mice. P = 0.5.

**Figure 5:**
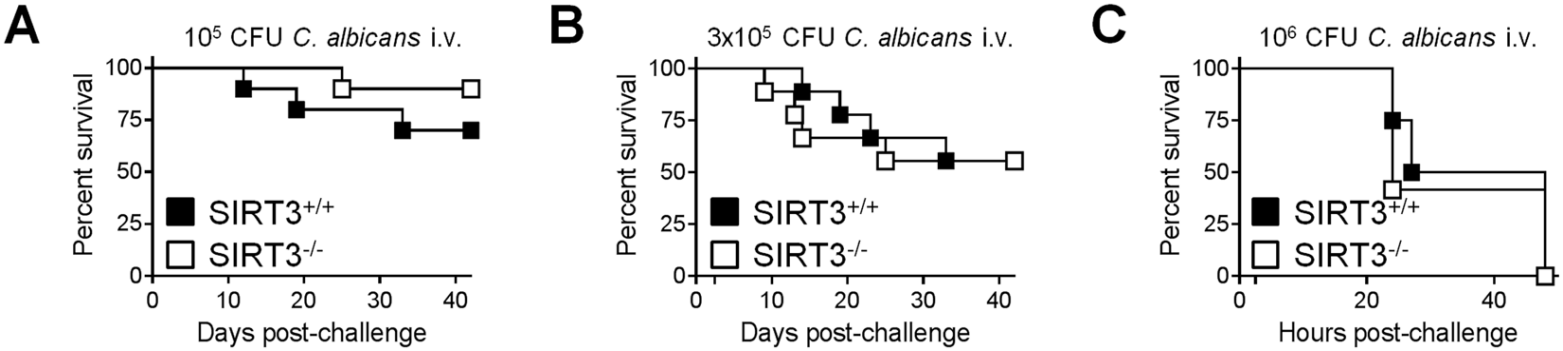
SIRT3 deficiency does not impact on survival of mice infected with *C*. *albicans*. SIRT3^+/+^ and SIRT3^−/−^ mice were injected i.v. with 10^5^ (**A**) n = 10 per group), 3 × 10^5^ (**B**) n = 9 per group) and 10^6^ CFU *C*. *albicans* (**C**) n = 12 per group). (**A**–**C**) Survival of mice. P = 0.3, 0.8 and 0.5.

We next examined the contribution of SIRT3 to host defenses against bacterial pneumonia and peritonitis, listeriosis and candidiasis. Infection models of diverse severity were used considering that SIRT3-mediated hypo-inflammatory response would jeopardize survival to otherwise non-lethal infection, while SIRT3-mediated hyper-inflammatory response would worsen outcome during severe infection^47, 51^. SIRT3^+/+^ and SIRT3^−/−^ mice survived equivalently to non-severe pneumonia induced by *K*. *pneumoniae* (82.5% *vs* 93%, P = 0.4, Fig. 3C). In a model of fulminant *E*. *coli* peritonitis, where all deaths occurred within 3 days, bacterial loads in blood collected 18 hours post-infection (median: 4.7 × 10^6^ CFU/ml *vs* 5.2 × 10^6^ CFU/ml; P = 0.4) and survival (27% *vs* 27%) were identical in SIRT3^+/+^ and SIRT3^−/−^ mice (Fig. 3D and E). Listeriosis was induced by i.v. challenge with the intracellular bacterium *L*. *monocytogenes*. Two days post-infection, bacteremia was low but slightly higher in SIRT3^−/−^ mice (median: 7.5 × 10^2^ CFU/ml *vs* 1.1 × 10^3^ CFU/ml; P = 0.02). *L*. *monocytogenes* burden in spleen and liver was massive but not significantly different between SIRT3^+/+^ and SIRT3^−/−^ mice (3.6 × 10^6^ CFU/mg *vs* 4.9 × 10^6^ CFU/mg, P = 0.8 and 1.3 × 10^5^ CFU/mg *vs* 1.8 × 10^5^ CFU/mg, P = 0.1; Fig. 4A). Death occurred between days 2 and 7, and overall survival was not influenced by SIRT3 deficiency (0% *vs* 7%, P = 0.5, Fig. 4B). Finally, candidiasis was induced by inoculating 10^5^, 3 × 10^5^ or 10^6^ CFU *C*. *albicans* to produce a mild/chronic infection inducing animal death over a period of 5 weeks or a severe infection leading to animal death within 3 days. In the three models, the survival rates of SIRT3^+/+^ and SIRT3^−/−^ mice were comparable (90% *vs* 70%, P = 0.3; 56% *vs* 56%, P = 0.8 and 0% *vs* 0%, P = 0.8) (Fig. 5A–C).

## Discussion

We report that SIRT3 deficiency has no major impact on immune cell development and host defenses against bacterial and fungal infections. These observations are particularly topical considering the promises of SIRT3-targeting strategies to treat age-related disorders.

SIRT3 deficiency did not alter the composition of the main lymphoid and DC subsets in thymus and spleen, in line with a previous study showing normal thymic, splenic and lymph node CD4^+^ and CD8^+^ T-cell subpopulations in SIRT3^−/−^ mice, including CD4^+^ Foxp3^+^ T regulatory cells (Tregs)^29^. Immune cells exposed to microbial products or cytokines undergo metabolic reprogramming characterized by a switch from oxidative phosphorylation to glycolysis associated with the development of inflammatory and antimicrobial functions^59^. Considering that SIRT3 impacts on mitochondrial biogenesis and functions^60^, we expected SIRT3 deficiency to enhance immune cell response to microbial stimulation. MAPK phosphorylation was reduced to some extend early on after LPS stimulation in SIRT3^−/−^ BMDMs. However, proliferation and cytokine production by splenocytes, macrophages and DCs were largely unaffected by SIRT3 deficiency. In contrast, shRNA-mediated SIRT3 silencing increased baseline TNF mRNA levels in RAW 264.7 macrophages^61^, and adenovirus-mediated SIRT3 overexpression inhibited MAPK phosphorylation in phenylephrine-treated myocytes and palmitate-stimulated pancreatic beta-cells^62, 63^. Thus, SIRT3 seems to have cell and possibly context-dependent effects. Supporting this assumption, SIRT3 deficiency impaired *in vitro* the suppressive function of Tregs, which are particularly dependent on SIRT3-mediated mitochondrial activity and oxidative phosphorylation to develop optimal functions. However, SIRT3^−/−^ Tregs retained their suppressive functions in an adoptive transfer model of cardiac allograft rejection^29^. SIRT3 deficiency also affected endothelial function in mice fed with a high cholesterol diet but not a normal diet^64^, and organ-specific SIRT3 deficiency increased mitochondrial protein acetylation but did not induce mitochondrial dysfunction and did not impact on overall metabolic homeostasis as observed in germline SIRT3 knockouts^43, 65^.

To assess the safety of SIRT3-targeting therapies, it was most important to analyze the contribution of SIRT3 in preclinical models of infection. As a first approach, we tested a model of endotoxemia, which revealed that cytokine response and survival rates were not different in SIRT3^+/+^ and SIRT3^−/−^ mice. Although SIRT3^−/−^ mice survived slightly better than SIRT3^+/+^ mice, whether this was a genuine effect would require large groups of animals (>40 mice per genotype) according to power calculation. Albeit very unlikely, we also cannot totally rule out that some differences in the genetic background of SIRT3^+/+^ and SIRT3^−/−^ mice play a role. To address that question, SIRT3^−/−^ mice with additional backcrosses should be tested. SIRT3^+/+^ mice had a minor yet significant survival advantage (10% *vs* 0% survival in SIRT3^+/+^ *vs* SIRT3^−/−^ mice) in a highly stringent model of endotoxemia^38^, suggesting that SIRT3 may provide benefits in sterile, deep inflammatory, processes. Unfortunately, the cytokine response was not reported. Endotoxemia does not recapitulate the complexity to host defense mechanisms generated to fight against living microorganisms. Moreover, immunomodulatory compounds may interfere with innate immune responses and compromise host defenses, as well documented for anti-TNF and anti-IL-1 agents^66, 67^. Thus, we elected to test models of sepsis induced by *E*. *coli* and *K*. *pneumoniae*, two of the most frequent etiologic agents of human sepsis. SIRT3 deficiency did not impact on the development of sub-acute pneumoniae and acute peritonitis, going well along with normal *in vitro* responses to bacterial stimulation of immune cells. In line with our observations, the survival rates of SIRT3^+/+^ and SIRT3^−/−^ mice were not significantly different following cecal ligation and puncture sepsis^22^. Additionally, SIRT3^+/+^ and SIRT3^−/−^ mice behaved roughly identically following systemic infection with *L*. *monocytogenes* and *C*. *albicans* given to produce chronic/mild and acute candidiasis. Interestingly, *Listeria* loads were slightly increased in the blood of SIRT3^−/−^ mice, which might feature transient alteration of mitochondrial dynamics during *L*. *monocytogenes* infection^68^. However, bacteremia was very low when compared to liver and spleen bacterial burdens, which were not different between SIRT3^+/+^ and SIRT3^−/−^ mice. The absence of patent phenotype in a panel of sepsis models suggests that infection-induced phagocyte recruitment and/or activity was not impaired in SIRT3^−/−^ mice. Indeed, SIRT3 deficiency did not affect endothelial activation, plaque macrophage and T cell infiltration and atherosclerosis in low-density lipoprotein receptor knockout mice^64^. Our observations are somehow reminiscent of that obtained analyzing SIRT1. While SIRT1 was globally shown to inhibit inflammation^69^, it had little impact on macrophage and neutrophil antimicrobial functions, and myeloid deficiency in SIRT1 did not influence the outcome of endotoxemia and Gram-positive sepsis^70^.

SIRT3 activity is strongly associated with metabolism, and there is a tight relationship between metabolism and immune functions^59^. Thus, work will be required to address whether SIRT3 impacts on host defenses under metabolic stress. It is also possible that sirtuins have complementary or redundant effects, as suggested by protein interaction studies^5^. Therefore, future studies should analyze the impact of targeting multiple sirtuins on innate immune responses. Supporting this strategy, dual inhibitors of SIRT1/2 and pan-classical HDAC inhibitors affected host defenses against infections^46, 51, 56, 71^. Considering that SIRT3 has been associated with age-related dysfunctions and that immune functions are decreased in elderly^72^, one should analyze the impact of SIRT3 in populations of different ages. Finally, a limitation of this study is that preclinical mouse models were performed with female mice. In a preliminary experiment using a limited number of males (seven animals), the survival of SIRT3^+/+^ and SIRT3^−/−^ mice to *Klebsiella*-induced pneumonia was not different. However, larger groups of mice and additional models should be tested to settle whether there is or not a sex-dependent impact of SIRT3 deficiency on susceptibility to infection.

Overall, our data support the assumption that SIRT3 has no major impact on innate immune functions and host defenses against bacterial and fungal infections, at least in healthy immunocompetent hosts. The present data largely support the safety of SIRT3-oriented therapies, in terms of susceptibility to infections, for treating metabolic, oncologic and neurodegenerative diseases.

## Electronic supplementary material

Antibodies used for flow cytometry analyses and full-length blots.

## References

[1] Savva A, Roger T (2013). Targeting toll-like receptors: promising therapeutic strategies for the management of sepsis-associated pathology and infectious diseases. Front Immunol.

[2] Sauve AA (2001). Chemistry of gene silencing: the mechanism of NAD+ -dependent deacetylation reactions. Biochemistry.

[3] Rauh D (2013). An acetylome peptide microarray reveals specificities and deacetylation substrates for all human sirtuin isoforms. Nat Commun.

[4] Lombard DB (2007). Mammalian Sir2 homolog SIRT3 regulates global mitochondrial lysine acetylation. Mol Cell Biol.

[5] Yang, W. *et al*. Mitochondrial Sirtuin Network Reveals Dynamic SIRT3-Dependent Deacetylation in Response to Membrane Depolarization. *Cell* **167**, 985–1000 e1021, doi:10.1016/j.cell.2016.10.016 (2016). 10.1016/j.cell.2016.10.016 PMC5134900 27881304

[6] Gurd BJ, Holloway GP, Yoshida Y, Bonen A (2012). In mammalian muscle, SIRT3 is present in mitochondria and not in the nucleus; and SIRT3 is upregulated by chronic muscle contraction in an adenosine monophosphate-activated protein kinase-independent manner. Metabolism.

[7] Iwahara T, Bonasio R, Narendra V, Reinberg D (2012). SIRT3 functions in the nucleus in the control of stress-related gene expression. Mol Cell Biol.

[8] Peek CB (2013). Circadian clock NAD+ cycle drives mitochondrial oxidative metabolism in mice. Science.

[9] Shi T, Wang F, Stieren E, Tong Q (2005). SIRT3, a mitochondrial sirtuin deacetylase, regulates mitochondrial function and thermogenesis in brown adipocytes. J Biol Chem.

[10] Hallows WC (2011). Sirt3 promotes the urea cycle and fatty acid oxidation during dietary restriction. Mol Cell.

[11] Hirschey MD (2010). SIRT3 regulates mitochondrial fatty-acid oxidation by reversible enzyme deacetylation. Nature.

[12] Shimazu T (2010). SIRT3 deacetylates mitochondrial 3-hydroxy-3-methylglutaryl CoA synthase 2 and regulates ketone body production. Cell Metab.

[13] Jing E (2013). Sirt3 regulates metabolic flexibility of skeletal muscle through reversible enzymatic deacetylation. Diabetes.

[14] Yu W (2016). Loss of SIRT3 Provides Growth Advantage for B Cell Malignancies. J Biol Chem.

[15] Someya S (2010). Sirt3 mediates reduction of oxidative damage and prevention of age-related hearing loss under caloric restriction. Cell.

[16] Bause AS, Haigis MC (2013). SIRT3 regulation of mitochondrial oxidative stress. Exp Gerontol.

[17] Albani D (2014). Modulation of human longevity by SIRT3 single nucleotide polymorphisms in the prospective study “Treviso Longeva (TRELONG)”. Age (Dordr).

[18] Bellizzi D (2005). A novel VNTR enhancer within the SIRT3 gene, a human homologue of SIR2, is associated with survival at oldest ages. Genomics.

[19] Lescai F (2009). Human longevity and 11p15.5: a study in 1321 centenarians. Eur J Hum Genet.

[20] TenNapel MJ (2014). SIRT6 minor allele genotype is associated with >5-year decrease in lifespan in an aged cohort. PLoS One.

[21] Koentges C, Bode C (2016). & Bugger, H. SIRT3 in Cardiac Physiology and Disease. Front Cardiovasc Med.

[22] Lu Y (2016). SIRT3 in cardiovascular diseases: Emerging roles and therapeutic implications. Int J Cardiol.

[23] McDonnell E, Peterson BS, Bomze HM, Hirschey MD (2015). SIRT3 regulates progression and development of diseases of aging. Trends Endocrinol Metab.

[24] Alhazzazi TY, Kamarajan P, Verdin E, Kapila YL (2011). SIRT3 and cancer: tumor promoter or suppressor?. Biochim Biophys Acta.

[25] Liu L, Peritore C, Ginsberg J, Kayhan M, Donmez G (2015). SIRT3 attenuates MPTP-induced nigrostriatal degeneration via enhancing mitochondrial antioxidant capacity. Neurochem Res.

[26] Weir HJ (2012). CNS SIRT3 expression is altered by reactive oxygen species and in Alzheimer’s disease. PLoS One.

[27] Yang W (2015). Mitochondrial Sirt3 Expression is Decreased in APP/PS1 Double Transgenic Mouse Model of Alzheimer’s Disease. Neurochem Res.

[28] Chalkiadaki A, Guarente L (2015). The multifaceted functions of sirtuins in cancer. Nat Rev Cancer.

[29] Beier UH (2015). Essential role of mitochondrial energy metabolism in Foxp3(+) T-regulatory cell function and allograft survival. FASEB J.

[30] Yu W (2016). Sirt3 deficiency exacerbates diabetic cardiac dysfunction: Role of Foxo3A-Parkin-mediated mitophagy. Biochim Biophys Acta.

[31] Gao J (2016). Deacetylation of MnSOD by PARP-regulated SIRT3 protects retinal capillary endothelial cells from hyperglycemia-induced damage. Biochem Biophys Res Commun.

[32] Lantier L (2015). SIRT3 Is Crucial for Maintaining Skeletal Muscle Insulin Action and Protects Against Severe Insulin Resistance in High-Fat-Fed Mice. Diabetes.

[33] Morigi M (2015). Sirtuin 3-dependent mitochondrial dynamic improvements protect against acute kidney injury. J Clin Invest.

[34] Zhao WY, Zhang L, Sui MX, Zhu YH, Zeng L (2016). Protective effects of sirtuin 3 in a murine model of sepsis-induced acute kidney injury. Sci Rep.

[35] Akamata, K. *et al*. SIRT3 is attenuated in systemic sclerosis skin and lungs, and its pharmacologic activation mitigates organ fibrosis. *Oncotarget*, doi:10.18632/oncotarget.12504 (2016). 10.18632/oncotarget.12504 PMC5342480 27732568

[36] Bindu, S. *et al*. SIRT3 blocks myofibroblast differentiation and pulmonary fibrosis by preventing mitochondrial DNA damage. *Am J Physiol Lung Cell Mol Physiol*, ajplung 00188 02016, doi:10.1152/ajplung.00188.2016 (2016). 10.1152/ajplung.00188.2016 PMC5283928 27815257

[37] Sosulski, M. L., Gongora, R., Feghali-Bostwick, C., Lasky, J. A. & Sanchez, C. G. Sirtuin 3 Deregulation Promotes Pulmonary Fibrosis. *J Gerontol A Biol Sci Med Sci*, doi:10.1093/gerona/glw151 (2016). 10.1093/gerona/glw151 PMC5964739 27522058

[38] Zeng H (2016). LPS causes pericyte loss and microvascular dysfunction via disruption of Sirt3/angiopoietins/Tie-2 and HIF-2alpha/Notch3 pathways. Sci Rep.

[39] Chen Y (2014). Sirtuin-3 (SIRT3), a therapeutic target with oncogenic and tumor-suppressive function in cancer. Cell Death Dis.

[40] Villalba JM, Alcain FJ (2012). Sirtuin activators and inhibitors. Biofactors.

[41] Liu TF (2015). Sequential actions of SIRT1-RELB-SIRT3 coordinate nuclear-mitochondrial communication during immunometabolic adaptation to acute inflammation and sepsis. J Biol Chem.

[42] Ren JH (2016). Protective Role of Sirtuin3 (SIRT3) in Oxidative Stress Mediated by Hepatitis B Virus X Protein Expression. PLoS One.

[43] Fernandez-Marcos PJ (2012). Muscle or liver-specific Sirt3 deficiency induces hyperacetylation of mitochondrial proteins without affecting global metabolic homeostasis. Sci Rep.

[44] Ciarlo E (2016). Impact of the microbial derived short chain fatty acid propionate on host susceptibility to bacterial and fungal infections *in vivo*. Sci Rep.

[45] Ciarlo E, Roger T (2016). Screening the Impact of Sirtuin Inhibitors on Inflammatory and Innate Immune Responses of Macrophages and in a Mouse Model of Endotoxic Shock. Methods Mol Biol.

[46] Mombelli M (2011). Histone deacetylase inhibitors impair antibacterial defenses of macrophages. J Infect Dis.

[47] Roger T (2009). Protection from lethal gram-negative bacterial sepsis by targeting Toll-like receptor 4. Proc Natl Acad Sci USA.

[48] Roger T (2016). High expression levels of macrophage migration inhibitory factor sustain the innate immune responses of neonates. Proc Natl Acad Sci USA.

[49] Savva A (2016). Functional polymorphisms of macrophage migration inhibitory factor as predictors of morbidity and mortality of pneumococcal meningitis. Proc Natl Acad Sci USA.

[50] Roger T, Ding X, Chanson AL, Renner P, Calandra T (2007). Regulation of constitutive and microbial pathogen-induced human macrophage migration inhibitory factor (MIF) gene expression. Eur J Immunol.

[51] Roger T (2011). Histone deacetylase inhibitors impair innate immune responses to Toll-like receptor agonists and to infection. Blood.

[52] Giannoni E (2011). Estradiol and progesterone strongly inhibit the innate immune response of mononuclear cells in newborns. Infect Immun.

[53] Piette C (2009). The dexamethasone-induced inhibition of proliferation, migration, and invasion in glioma cell lines is antagonized by macrophage migration inhibitory factor (MIF) and can be enhanced by specific MIF inhibitors. J Biol Chem.

[54] Lugrin J (2009). Histone deacetylase inhibitors repress macrophage migration inhibitory factor (MIF) expression by targeting MIF gene transcription through a local chromatin deacetylation. Biochim Biophys Acta.

[55] Perreau M (2014). Exhaustion of bacteria-specific CD4 T cells and microbial translocation in common variable immunodeficiency disorders. J Exp Med.

[56] Lugrin J (2013). The sirtuin inhibitor cambinol impairs MAPK signaling, inhibits inflammatory and innate immune responses and protects from septic shock. Biochim Biophys Acta.

[57] Vacher G (2015). Innate Immune Sensing of Fusarium culmorum by Mouse Dendritic Cells. J Toxicol Environ Health A.

[58] Roger T (2013). Macrophage migration inhibitory factor deficiency is associated with impaired killing of gram-negative bacteria by macrophages and increased susceptibility to Klebsiella pneumoniae sepsis. J Infect Dis.

[59] O’Neill LA, Pearce EJ (2016). Immunometabolism governs dendritic cell and macrophage function. J Exp Med.

[60] Giralt A, Villarroya F (2012). SIRT3, a pivotal actor in mitochondrial functions: metabolism, cell death and aging. Biochem J.

[61] Xu H, Hertzel AV, Steen KA, Bernlohr DA (2016). Loss of Fatty Acid Binding Protein 4/aP2 Reduces Macrophage Inflammation Through Activation of SIRT3. Mol Endocrinol.

[62] Sundaresan NR (2009). Sirt3 blocks the cardiac hypertrophic response by augmenting Foxo3a-dependent antioxidant defense mechanisms in mice. J Clin Invest.

[63] Kim M (2015). SIRT3 Overexpression Attenuates Palmitate-Induced Pancreatic beta-Cell Dysfunction. PLoS One.

[64] Winnik S (2014). Deletion of Sirt3 does not affect atherosclerosis but accelerates weight gain and impairs rapid metabolic adaptation in LDL receptor knockout mice: implications for cardiovascular risk factor development. Basic Res Cardiol.

[65] Lombard DB, Zwaans BM (2014). SIRT3: as simple as it seems?. Gerontology.

[66] Lopalco G (2016). Safety profile of anakinra in the management of rheumatologic, metabolic and autoinflammatory disorders. Clin Exp Rheumatol.

[67] Murdaca G (2015). Infection risk associated with anti-TNF-alpha agents: a review. Expert Opin Drug Saf.

[68] Stavru F, Bouillaud F, Sartori A, Ricquier D, Cossart P (2011). Listeria monocytogenes transiently alters mitochondrial dynamics during infection. Proc Natl Acad Sci USA.

[69] Preyat N, Leo O (2013). Sirtuin deacylases: a molecular link between metabolism and immunity. J Leukoc Biol.

[70] Crotty Alexander LE (2013). Myeloid cell sirtuin-1 expression does not alter host immune responses to Gram-negative endotoxemia or Gram-positive bacterial infection. PLoS One.

[71] Ciarlo E, Savva A, Roger T (2013). Epigenetics in sepsis: targeting histone deacetylases. Int J Antimicrob Agents.

[72] Shaw AC, Goldstein DR, Montgomery RR (2013). Age-dependent dysregulation of innate immunity. Nat Rev Immunol.

