# Supplementary material for: Sirtuin 3 deficiency does not alter host defenses against bacterial and fungal infections
Source: Sci Rep. 2017 Jun 20;7:3853. doi: 10.1038/s41598-017-04263-x (PMC5478639; doi:10.1038/s41598-017-04263-x)
Supplement: Supplementary file 1 — Antibodies used for flow cytometry analyses and full-length blots. [file 41598_2017_4263_MOESM1_ESM.pdf]

Sirtuin 3 deficiency does not alter host defenses against bacterial and fungal infections

Eleonora Ciarlo, Tytti Heinonen, Jérôme Lugrin, Hans Acha-Orbea, Didier Le Roy, Johan Auwerx, Thierry Roger

Supplementary Table S1. Antibodies used for flow cytometry analyses

**Target**	**Clone name**	**Coupling**
B220	RA3-6B2	eFluor® 450
CD3	145-2C11	PE, eFluor® 450
CD4	RM4-5	PE, FITC
CD8	53-6.7	APC-eFluor® 780, APC-Cy7
CD11b	M1/710	PE, APC
CD11c	HL3	APC
CD23	B3B4	PE
CD25	PC61.5	APC
CD44	IM7	APC, eFluor® 450
CD62L	MEL-14	FITC
IgD	AMS 9.1	FITC
MHC-II	114.15.2	FITC

PE: phycoerythrin; FITC: fluorescein isothiocyanate; APC: allophycocyanin. All antibodies were from eBioscience, except the anti-IgD-FITC that was from BD Biosciences.

Supplementary Figure S1

Full-length blots

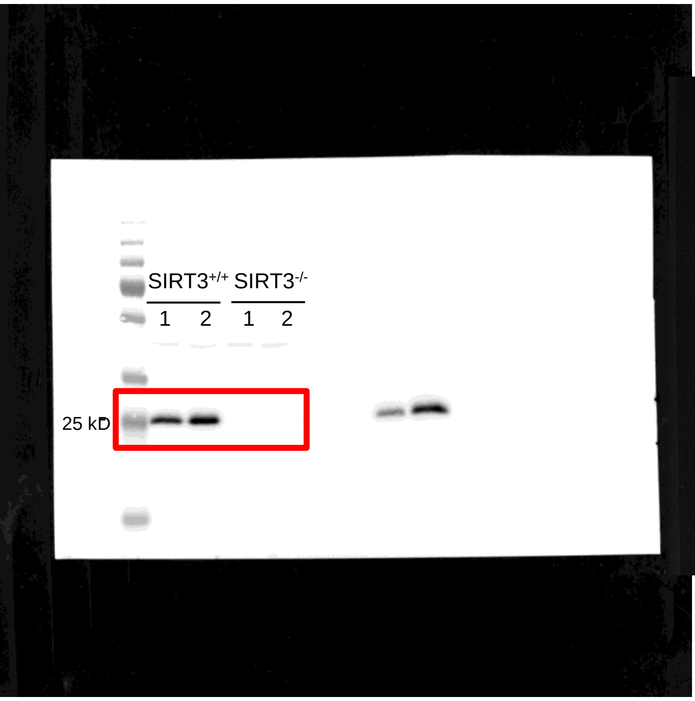

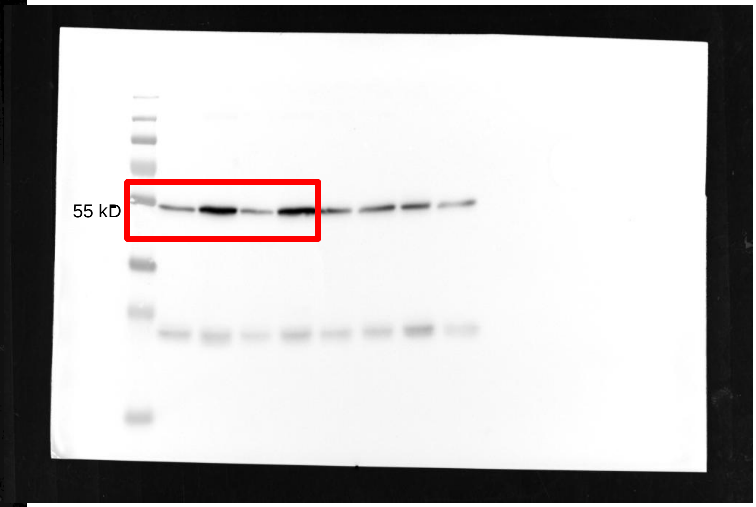

Panels used in Figure 1 C

Supplementary Figure S2

Full-length blots

Western blots
phospho-ERK1/2

Western blots
total ERK1/2

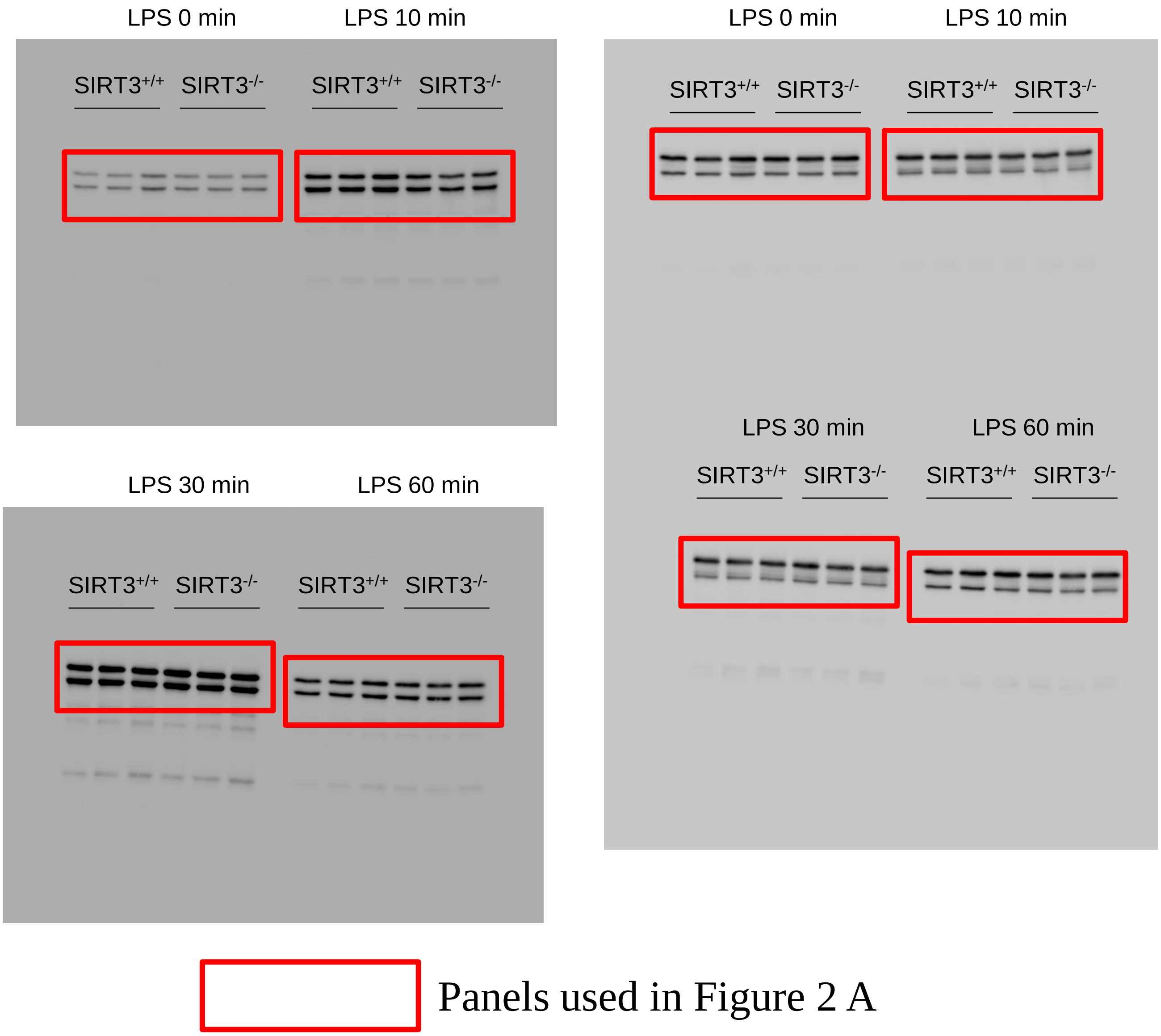

Supplementary Figure S3

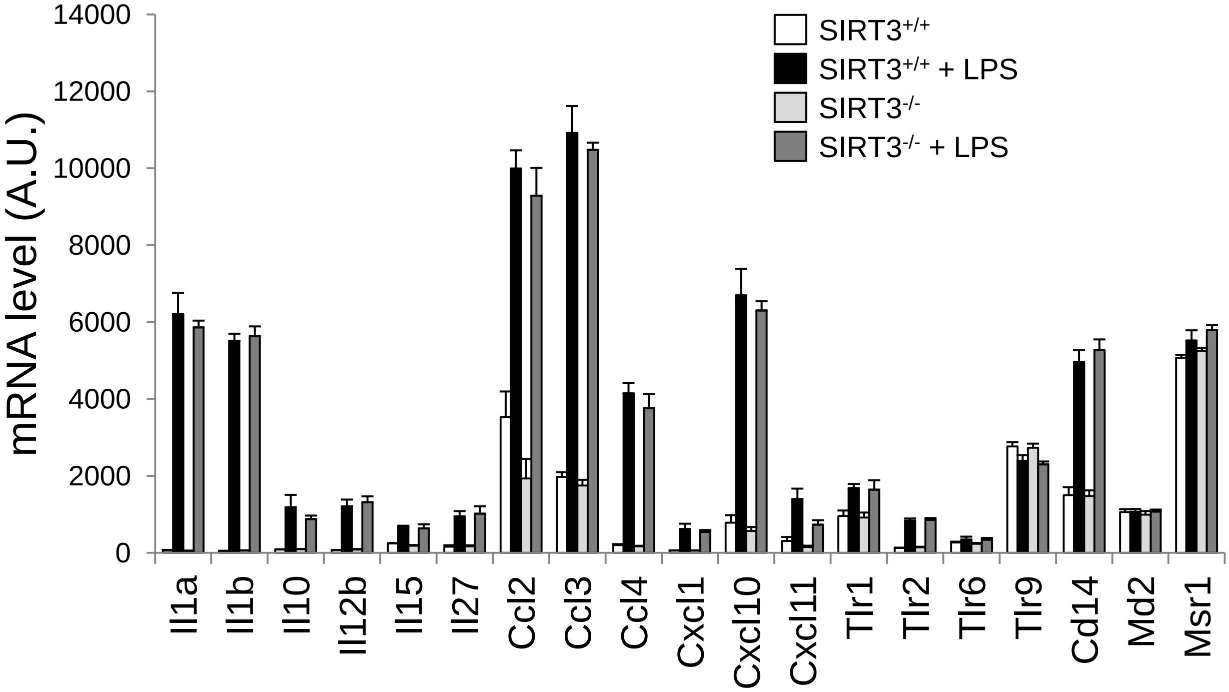

SIRT3 does not affect the expression of cytokines/chemokines and PRRs. Il1a, Il1b, Il10, Il12b, Il15, Il27, Ccl2/Mcp1, Ccl3/Mip1a, Ccl4/Mip1b, Cxcl1/Groa, Cxcl10/Ip10, Cxcl11/Itac, Tlr1, Tlr2, Tlr6, Tlr9, Cd14, Md2 and Msr1 mRNA expression levels in SIRT3$^{+/+}$ and SIRT3$^{-/-}$ BMDMs incubated for 2 hours with or without LPS (10 ng/ml). Data are means ± SD from one experiment performed with 3 mice. A.U.: arbitrary units.
